# Molecular epidemiology and genotype-specific disease severity of hepatitis E virus infections in Germany, 2010–2019

**DOI:** 10.1080/22221751.2022.2091479

**Published:** 2022-07-17

**Authors:** Mathias Schemmerer, Jürgen J. Wenzel, Klaus Stark, Mirko Faber

**Affiliations:** a National Consultant Laboratory for HAV and HEV, Institute of Clinical Microbiology and Hygiene, University Medical Center Regensburg, Regensburg, Germany; b Department for Infectious Disease Epidemiology, Robert Koch Institute, Berlin, Germany

**Keywords:** Hepatitis E virus, Orthohepevirus A, molecular epidemiology, pathogenicity, foodborne infection, zoonosis, disease outbreaks

## Abstract

Zoonotic hepatitis E virus (HEV) is endemic in Europe. Genotype 3 (HEV-3) is predominant but information on subtype distribution, trends and clinical implications in Germany is scarce. We analysed 936 HEV RNA positive samples of human origin and corresponding national surveillance data from 2010 to 2019. Samples were referred to the National Consultant Laboratory and sequenced in at least one of four genomic regions. Sequences were analysed using bioinformatics methods and compared to the latest HEV reference set. 1,656 sequences were obtained from 300 female, 611 male and 25 of unknown sex aged 3–92 years (median 55 years). HEV-3c was predominant (67.3%) followed by HEV-3f, HEV-3e and HEV-3i(-like) with 14.3%, 9.7% and 4.0% (other subtypes ≤1.1%). The proportion of HEV-3 group 2 (3abchijklm) strains increased over time. Jaundice, upper abdominal pain, fever, hospitalization, and death due to HEV were significantly more often reported for patients infected with HEV-3 group 1 (3efg) compared to group 2. Larger spatio-temporal clusters of identical sequences were not observed. HEV-3 group 1 infections are more severe as compared to the predominant group 2. Detection of group 2 strains increased over the last years, possibly due to more frequent diagnosis of asymptomatic and mild courses. The diversity of strains and the space–time distribution is compatible with a foodborne zoonosis with supra-regional distribution of the infection vehicle (pork products).

## GRAPHICAL ABSTRACT

**Figure:**
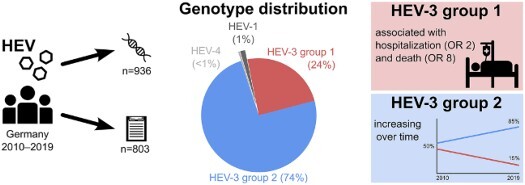

## Introduction

Hepatitis E virus (HEV) is phylogenetically divided into eight genotypes (HEV-1 to -8) [1]. HEV-1 and -2 solely infect humans and are mostly transmitted faecal-orally in developing countries through contaminated water, also causing large outbreaks [2]. In contrast, HEV-3 and -4 are predominantly transmitted zoonotically to humans via undercooked pork and pork products but also game meat [3]. HEV-5 and -6 were so far only detected in wild boars from Japan. HEV-7 and -8 have recently been detected in camels in the Middle East and China. Of the latter four genotypes, only one human infection with HEV-7 – attributed to consumption of contaminated camel meat and milk – has been described [4]. While HEV typically causes acute hepatitis, HEV-3 infections are often mild or asymptomatic and therefore remain undiagnosed [5]. Still, HEV infection represents the main cause of acute hepatitis in several European countries and chronification of HEV in immunocompromised patients can pose a severe risk [6]. Several EU/EEA member states have implemented HEV-specific surveillance systems and joint efforts for standardization are underway [7]. In Germany, the annual number of notified hepatitis E cases has exceeded those of hepatitis A in each year since 2015 (https://survstat.rki.de). High anti-HEV IgG prevalence rates of up to 52.5% have been reported from several European countries [8]. The respective studies repeatedly observed increasing seroprevalence rates with age. This effect can be seen in line with a very common alimentary exposure risk over an individual’s lifetime and the resulting cumulative effect on seropositivity rates by age. Based on seroincidence data, the annual number of infections in Germany was estimated at approximately 400,000 [9].

Three monophyletic clades are observed within HEV-3: group 1 (subtypes HEV-3e, f and g), group 2 (HEV-3a, b, c, h, i, j, k, l and m) and HEV-3ra (rabbit) [1]. Information on subtype distribution and dynamics in Europe is available for few countries. In England and Wales, a shift from HEV-3 group 1 to group 2 strains was observed between 2003 and 2012 [10]. A comparable shift was found in Belgium where HEV-3f used to be the most frequently detected subtype until 2015, while HEV-3c became the most common subtype in 2016 and 2017 [11]. In the Netherlands, the transition from 3f to 3c was observed in pigs while data from humans was only available from years after 3c already dominated [12]. A less pronounced decline in group 1 strains since 2003 has been observed in France, which is probably still ongoing as mainly 3f declined in favour of 3c but still dominated between 2010–2016 with 58.0% versus 22.6% [13–15]. An exception is Italy where HEV-3f remained the dominant subtype and no shift was observed neither in humans nor in pigs and wild boars [16]. These changes may be of clinical relevance but studies on the correlation between HEV-3 groups or subtypes and clinical courses yielded conflicting results [17–20] while infections of rabbits pointed to subtype-specific pathogenicity [21]. Data for Germany are scarce and either regionally limited or were published more than ten years ago [22,23].

Our objective was to analyse HEV geno- and subtype distribution in patients with hepatitis E in Germany from 2010 to 2019. Moreover, we investigated temporal trends, disease severity and spatio-temporal clusters of infection by linking phylogenetic with demographical and clinical data available from the national surveillance system.

## Materials and methods

### Sample collection and study design

Acute laboratory-confirmed HEV infections have been notifiable in Germany since 2001. Serological evidence or detection of viral RNA by reverse transcription-PCR (RT–PCR) is mandatorily reported to the local public health departments. The health departments complete and verify case information according to the national surveillance case definition. Information about onset, symptoms, hospitalization, outcome and probable place of infection is requested from the patient or treating physician. Case data are anonymized and electronically transmitted to the state health department and from there to the Robert Koch Institute, the national public health institute in Germany. Prior to and during the sampling period, public health departments had been informed about the intensified molecular surveillance programme through official information channels. Local public health departments or diagnostic laboratories were asked to refer patients’ specimen to the national consultant laboratory for further investigation of a clinical (e.g. jaundice, upper abdominal pain or elevated liver enzymes) or laboratory suspicion (reactive anti-HEV IgM) of hepatitis E. Samples typically consisted of residual serum, plasma or faeces taken for primary diagnostics in the acute phase. The investigations described in this article were conducted in the framework of surveillance activities according to §13 of the infection protection act. Approval by an ethics committee was thus not required.

### HEV RNA quantification

Nucleic acid was extracted from samples on an EZ1® Advanced XL workstation using the EZ1 Virus Mini Kit v2.0 (Qiagen, Hilden, Germany). From 2009 to 2015, HEV was detected by RT-qPCR according to Wenzel et al. [24]. In 2016, the RT-qPCR was replaced by a protocol by Jothikumar et al. with a modified probe [25,26]. Both assays were calibrated against the WHO International Standard (code number 6329/10) and HEV RNA was quantified as International Units per mL (IU/mL).

### HEV sequencing

Purified RNA was reverse-transcribed and amplified by a first round and consecutive nested PCR. Products were purified and sequenced on an ABI 3130xl sequencer. The resulting electropherograms were analysed and assembled with CodonCode Aligner v4.2.7 (www.codoncode.com, CodonCode Corporation, Centerville, MA, USA). HEV sequencing was started in 2010 with a 242 bp and a 178 bp fragment (primers excluded) in ORF1 and ORF2, respectively [24]. In 2016, sequencing of the ORF2 fragment was replaced by a nested broad-spectrum RT–PCR targeting a 280 bp RdRp fragment [27]. This was replaced in 2018 by the unified HEVnet protocol (https://www.rivm.nl/en/hevnet), which makes use of a set of degenerated first round and nested PCR primers to analyse a 493 bp ORF2 fragment. The location of the fragments in relation to the HEV-3c reference sequence is shown in Supplementary Figure 1. Sequence data from this article have been deposited with the International Nucleotide Sequence Database Collaboration Libraries (GenBank, DDBJ and ENA) under the accession numbers MZ813385 – MZ814966 and were analysed together with sequences published in earlier studies (accession numbers HG998145 – HG998188, FN985024–FN985026, FN994997, FN995000, FR687017, FR728243, FR728245-FR728256, FR846450–FR846452, HE605113–HE605117, HE716853, HE716854, HF912156, and HF912157).

### Genotyping and phylogenetic analysis

Sequence fragments were compared to the HEV reference set by Smith et al. 2020 using the fasta36 algorithm [1,28]. In case of multiple sequences per sample, results of the fragment with the highest bit score were used. For phylogenetic analyses, a core multiple sequence alignment was generated using the whole genome HEV reference strains. Then, sequence fragments were merged sample-specific to one 1193 bp long sequence per sample (ORF1 + RdRp + net + ORF2 = 242 bp + 280 bp + 493 bp + 178 bp) and aligned to the core alignment. This alignment was extended with moose HEV (accession KF951328) as an outgroup. Sequence alignments were created with MAFFT 7 and analysed phylogenetically with RAxML v8.2.12 [29,30]. The best matching consensus tree was calculated based on the maximum likelihood principle with a bootstrap of 1,000 replicates and visualized with FigTree 1.4.4 (http://tree.bio.ed.ac.uk/software/figtree/).

### Identification of identical sequences

In analogy to an established procedure in molecular hepatitis A virus (HAV) surveillance and outbreak detection, we compared sequences for high homology to identify related cases by searching fragment-specific sequence files using the fasta36 algorithm. For HAV, a confirmed outbreak case is defined with a sequence identity of  ≥ 99.4% to an outbreak strain, based on a 460 bp fragment (≤ 2 mismatches in 460 bp) [31]. Since there is not yet an established cut-off for HEV sequence deviation that would still count as outbreak-related, we chose to apply a more stringent definition: HEV strains had to be sequenced in the same two genomic regions and those two fragments had to be 100% identical. Although this approach might suffer in sensitivity in terms of missing related cases, we preferred to minimize the chance of producing false positives.

### Statistical analysis

Linear regression was performed for HEV-3 group 1 and 2 case numbers aggregated by year. Data were tested for normality using the Shapiro–Wilk test. Normally distributed data were further analysed with the *t*-test and one-way ANOVA for two and more groups. Not normally distributed data were analysed using the rank-sum test and ANOVA on ranks for two and more groups. Trend analyses were performed with the Cochran–Armitage test for trend. We used logistic regression analysis (complete case analysis) to assess the association of different HEV-3 subtypes (group 1 vs. group 2) with the presence of symptoms (fever, jaundice, upper abdominal pain and elevated liver enzymes), hospitalization and death. Age and sex of the patient as well as year and month of notification of the case was included in the model to adjust for possible confounding.

## Results

### Study population

**Figure 1.**
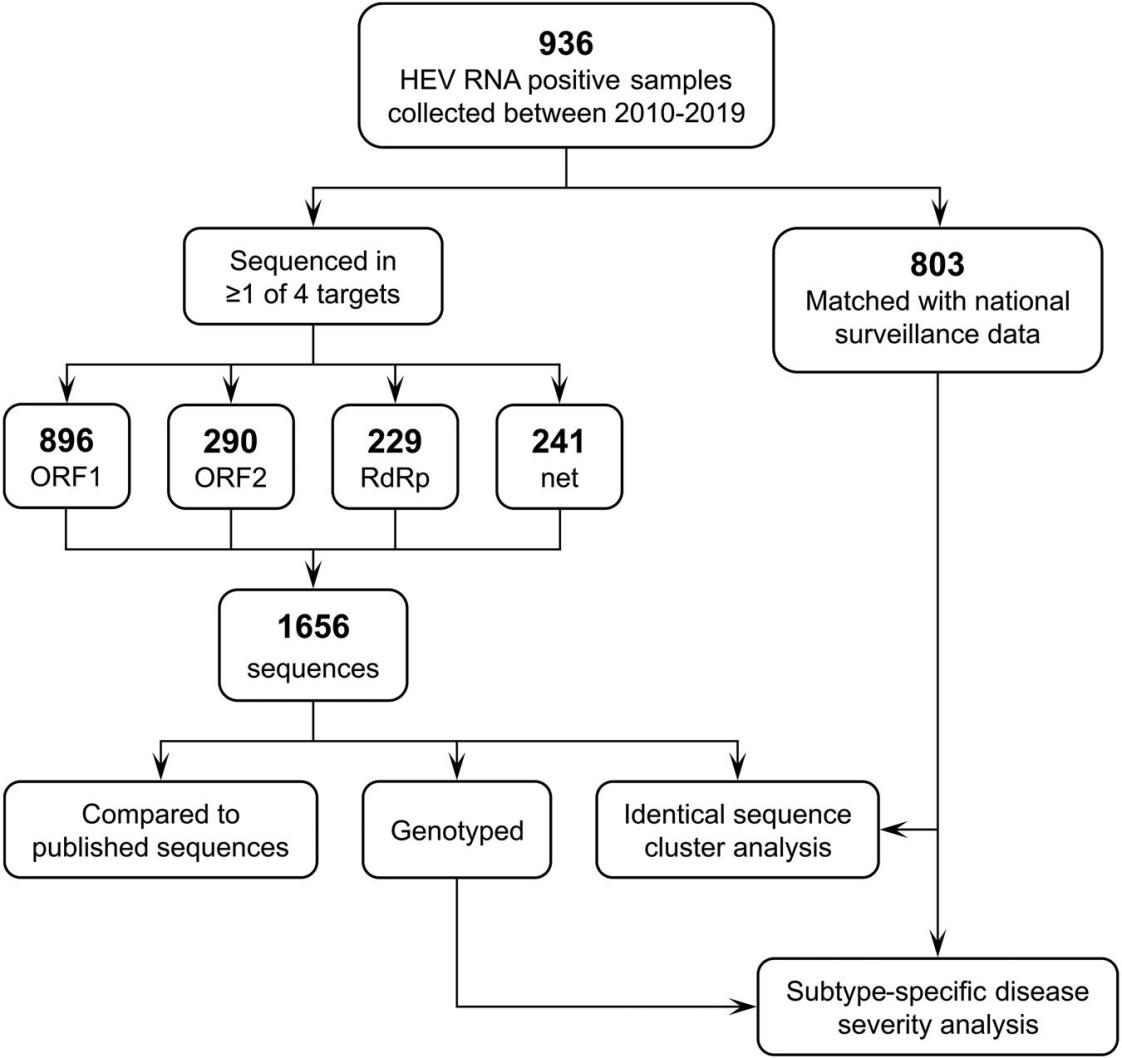
Study design. ORF, open reading frame; RdRp, RNA-dependent RNA polymerase; net, HEVnet unified sequencing protocol fragment.

1,656 sequences – comprising 896 ORF1, 290 ORF2, 229 RdRp and 241 net fragments – were obtained from 936 HEV PCR positive individuals during 2010–2019 (Figure 1). Of these, 803/936 (83.3%) could be matched with a case of HEV infection notified through the national infectious disease surveillance. Table 1 shows the study population by demographic and clinical characteristics.

**Table 1.** Study population by demographic and clinical characteristics, Germany, 2010–2019. “n” in brackets indicates number of individuals with available data for the respective characteristic.

Characteristic	*N*	%
Sex (*n* = 911)		
Female	300	32.9
Male	611	67.1
Age group (*n* = 888)		
≤9	5	0.6
10–19	25	2.8
20–29	39	4.4
30–39	84	9.5
40–49	150	16.9
50–59	235	26.5
60–69	179	20.2
70–79	133	15.0
≥80	38	4.3
Place of Residence (*n* = 803)		
North	52	6.5
West	315	39.2
East	217	27.0
South	219	27.3
Presence of symptoms (*n* = 718)		
Fever (≥ 38.5°C)	156	21.7
Jaundice	275	38.3
Upper abdominal pain	320	44.6
Elevated liver enzymes	516	64.3
Hospitalized (*n* = 733)		
Yes	501	68.4
No	232	31.7
Death due to hepatitis E (*n* = 781)		
Yes	7	0.9
No	774	99.1
Total	936	100.0

### Genotype and subtype distribution and dynamics

**Figure 2.**
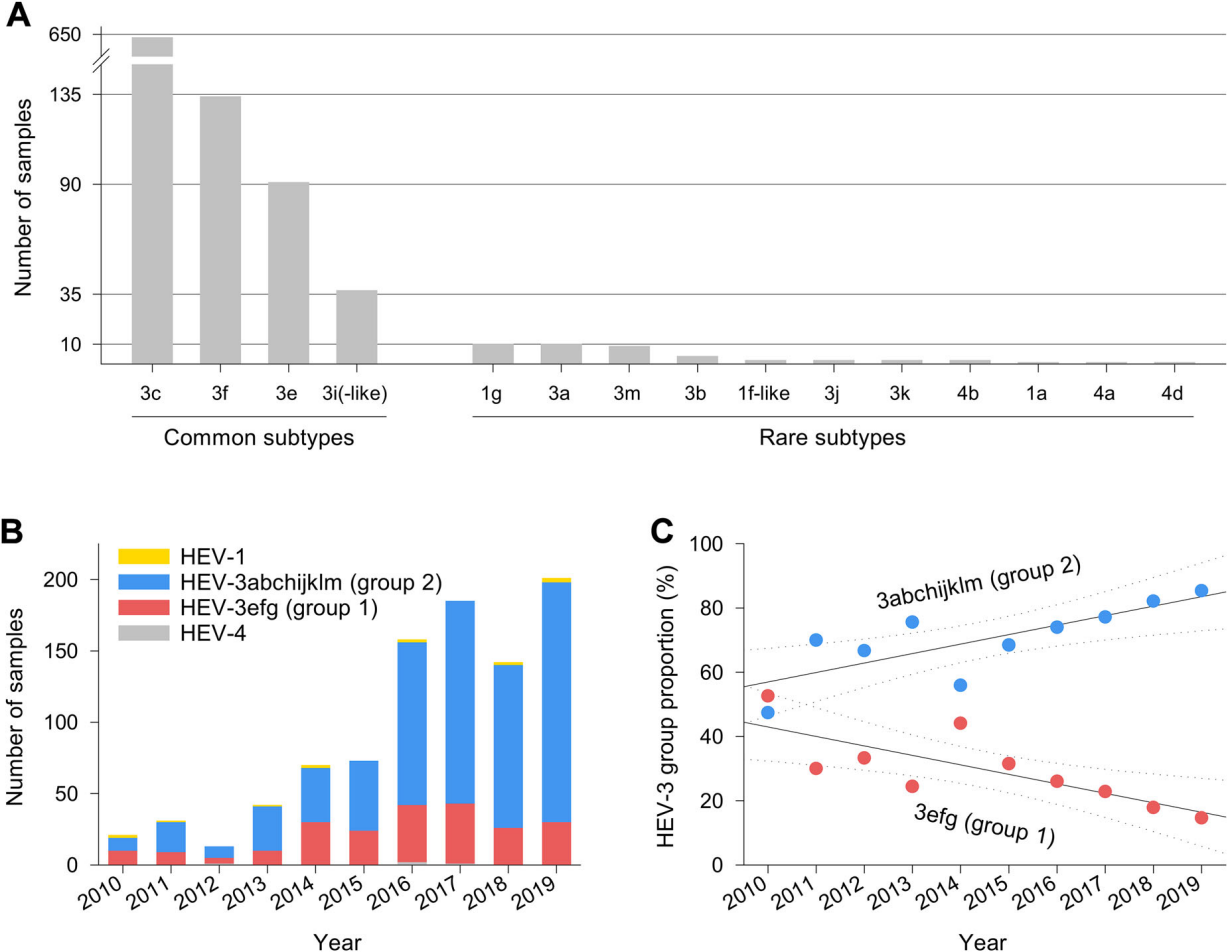
Distribution of samples referred to the national consultant laboratory. Samples depicted by (A) HEV subtypes, (B) year, genotype and HEV-3 group, and (C) year and HEV-3 group proportion (solid and dotted lines show linear regressions and 95% confidence intervals, respectively).

**Figure 3.**
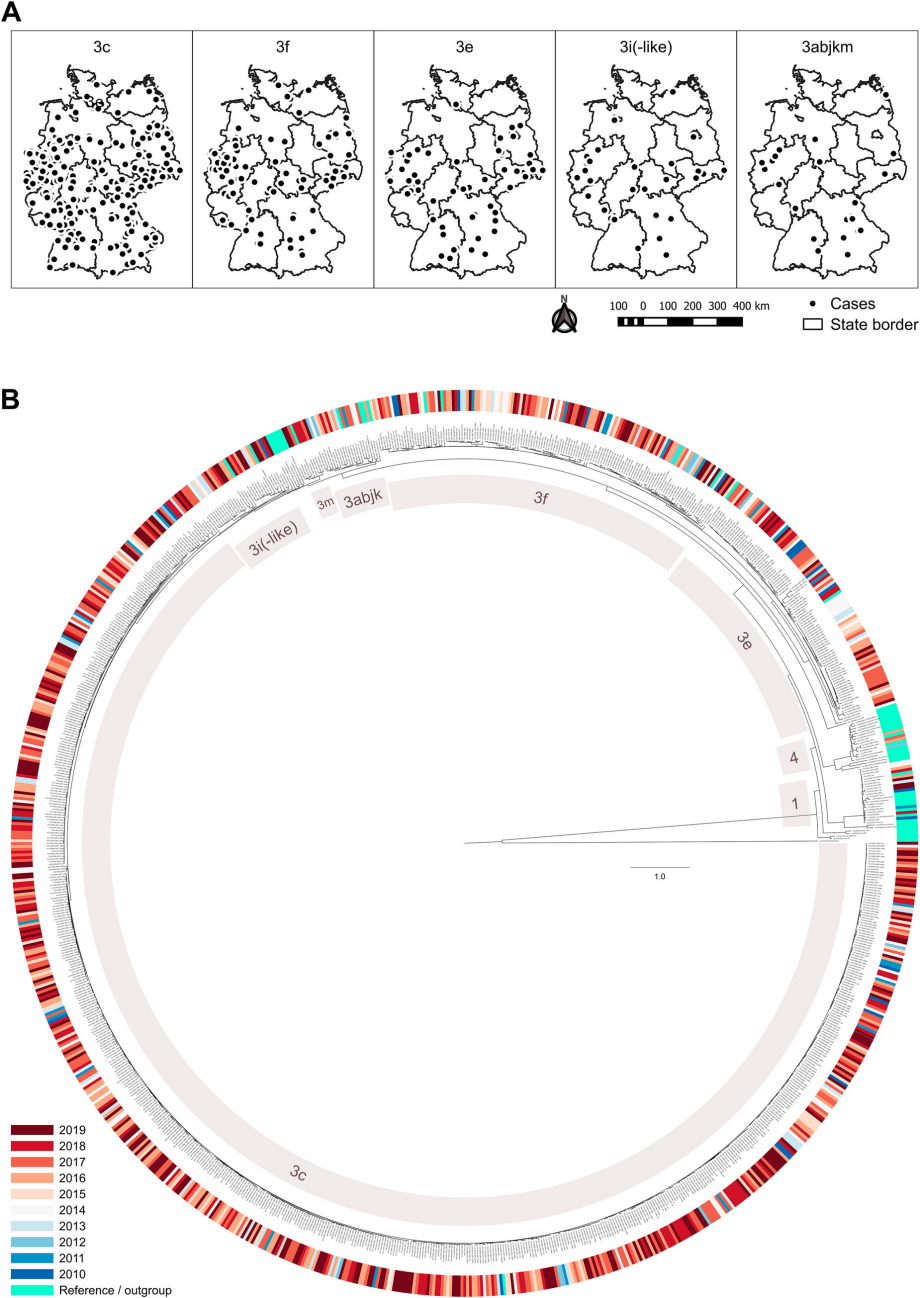
Overview of subtype occurrence by place and time. (A) Geographical distribution of HEV-3 subtypes and monophyletic clade 3abjkm in Germany, 2010–2019. Dots represent 5-digit-postal code districts with the occurrence of at least one case of the respective HEV subtype or clade. (B) Phylogenetic tree built with all sequences (*n* = 1656), which were merged sample-specific (*n* = 936) and extended with the current reference set. The best matching tree was calculated with RAxML, 1000 bootstrap replicates, and rooted with moose HEV (GenBank KF951328.2). HEV-3 subtypes as well as HEV-1 and -4 are labelled.

HEV-3c was the most frequent subtype, accounting for 67.3% of all infections, followed by HEV-3f, HEV-3e and HEV-3i(-like) with 14.3%, 9.7% and 4.0%, respectively. Subtypes HEV-3a, b, j, k and m, HEV-1a, f-like and g and HEV-4a, b and d were found in only 1–10 individuals each during the 10-year observation period (Figure 2(A)). Among patients with genotype 3 infections and with available demographic data, 600/896 (67.0%) were male and 50% were 55 years and older (interquartile range, IQR: 45–66 years). Genotype 1 infections (ten HEV-1 g, two HEV-1f-like, one HEV-1a) typically occurred in younger persons with a similar distribution of sexes (IQR: 17–34 years, 63.6% male) and a travel history to India, Pakistan or Bangladesh. Patients with genotype 4 infections (two HEV-4b, one HEV-4a, one HEV-4d) were between 33 and 51 years old, all were male and one (infected with HEV-4d) had a travel history to China. In line with the overall trend in notified hepatitis E cases in Germany, the number of HEV PCR positive samples analysed rose continuously between 2010 and 2019 (Figure 2(B)). While the annual number of detected HEV-1 and -4 remained constant on a very low level, the number of HEV-3 strains steadily increased. The proportion of HEV-3abchijklm (group 2) among all HEV-3 subtypes increased on average by approximately 3% per year, while HEV-3efg (group 1) decreased equally (Figure 2(C)). Generally, we observed hepatitis E cases in all parts of the country. Specific subtypes did not cluster according to an area of living or year of sample collection (Figure 3). We did not detect rabbit HEV sequences. Of note, we detected HEV-3m only in 2017–2019 (between two and three cases yearly, see Supplementary Table 1). We also observed a slight seasonality of HEV infections with higher numbers in spring and summer (Supplementary Figure 2).

### HEV-3 group-specific disease severity

In the phylogenetic analysis, genotype 3 sequences belonged to one of two monophyletic groups. Individuals with HEV-3 group 1 (3e and 3f) infections (225 of 919, 24.5%), compared to individuals with HEV-3 group 2 infections (694 of 919, 75.5%) were significantly more likely to present with jaundice (55.3% vs. 31.7%, *p* < 0.001), upper abdominal pain (52.0% vs. 42.2%, *p* = 0.031) or fever (26.8% vs. 19.2%, *p* = 0.023). They were also significantly more likely to be hospitalized (79.5% vs. 64.5%, *p* = 0.002). Similarly, death attributed to HEV-3 infection occurred in 5 of 182 (2.7%) of group 1 infections (three HEV-3e, two HEV-3f) and in 2 of 586 (0.3%) of group 2 infections (one HEV3-c and one HEV3-i-like), respectively. While there was no statistically significant difference in the proportion of males, mean age was higher in cases with group 1 infections compared to group 2 infections (57.1 vs. 54.1 years, *p* = 0.016). The differences in the frequency of symptoms, hospitalization and death remained statistically significant in a multivariable analysis adjusting for age, sex and time of notification. Results of uni- and multivariable analyses are shown in Table 2. No significant difference in the frequency of elevated liver enzymes or viral load was observed between individuals infected with HEV-3 group 1 and group 2 (Supplementary Figure 3).

**Table 2.** Absolute and relative frequency of symptoms by HEV-3 group and results of multivariable analysis (adjusted OR, 95% CI for HEV-3e and -3f).

Characteristic	HEV-3e & f		HEV-3a, b, c, i, j, k & m (group 2)		OR (95% CI)^a^	*P* value^a^
(group 1)	*n*	%	*n*	%		
Elevated liver enzymes	131/179	73.2	374/526	71.1	1.13 (0.77–1.68)	0.526
Jaundice	99/179	55.3	167/526	31.7	2.38 (1.66–3.41)	<0.001
Upper abdominal pain	93/179	52.0	222/526	42.2	1.47 (1.00–2.09)	0.031
Fever (≥ 38.5 °C)	48/179	26.8	101/526	19.2	1.61 (1.07–2.42)	0.023
Hospitalized	140/176	79.5	351/544	64.5	1.93 (1.27–2.94)	0.002
Death due to hepatitis E	5/182	2.7	2/586	0.3	7.61 (1.41–41.01)	0.018

Note: Notified hepatitis E cases with genotype 3 sequencing result, Germany, 2010–2019. a, adjusted by age, sex, year and month of notification; CI, confidence interval; OR, odds ratio.

### Identical sequence clusters

**Figure 4.**
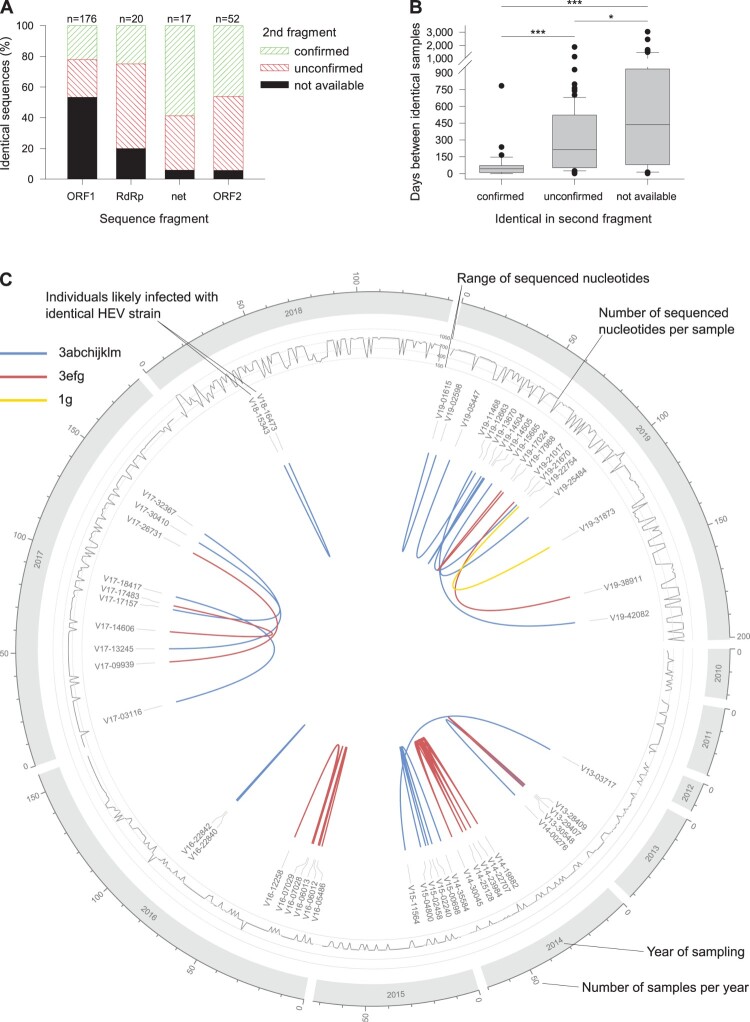
Analysis of samples with identical sequences. (A) Sequences were subject to fragment-specific fasta36 similarity searches. If available, identical fragments were double-checked with a second fragment. (B) Time between collection of samples with at least one identical sequence match. (C) Distribution of samples with two identical fragments. *p = 0.024; ***p < 0.001.

We identified 243 samples with sequence identity in at least one of the sequenced regions (226 pairwise sequence matches). A second sequence fragment was available for 124 sample pairs, of which in 39 (31.5%) and 85 (68.5%) the match was confirmed or not confirmed, respectively. The match distribution on fragment level is shown in Figure 4(A). The time between sample pairs was statistically significantly less in confirmed vs. unconfirmed matches (44 vs. 215 days, *p* < 0.001, Figure 4(B)). Given the high proportion of not identical second fragments (68.5%) and the significantly higher time between matched samples with one comparable fragment, only confirmed matches are shown throughout the observation period (Figure 4(C)). We identified 24 strains infecting more than one individual. Most clusters involved two individuals (*n* = 20) and rarely three (*n* = 3) and five (*n* = 1). The largest cluster involved 6 individuals and was caused by an outbreak of an HEV-3e strain in one county in 2014. Due to our strict criteria, however, only five individuals were counted, since no ORF2 fragment was available for case V14-14332 having an identical ORF1 fragment only. The median geographical distances and elapsed time of clusters were 120 km (range 0–480 km) and 51 days (range 0–782 days), respectively. No HEV-3 group-specific differences in these characteristics were observed. However, the proportion of HEV-3efg sequences was higher in clusters (33.3%) than in the total study population (24.0%). Two of seven subjects who died of the disease were also part of clusters: V14-30045 (HEV-3e) and V19-21017 (HEV-3f).

## Discussion

In this study, we investigated the phylogenetic and epidemiological situation of HEV in Germany between 2010 and 2019. We found HEV-3c to be the predominant subtype and a continuing increase in the proportion of HEV-3 group 2 (3abchijklm) strains. Infections with HEV-3 group 1 (3efg) were associated with a more severe course of disease, hospitalization and death. We did not observe subtype-specific geographical or temporal clustering.

The majority of HEV strains sequenced in Europe belong to subtypes 3c, 3f and 3e. Our study showed a clear dominance of subtype 3c in Germany, followed by 3f and 3e. This is comparable to the situation in England, Belgium and the Netherlands [11,12,32,33]. In France, 3f is still reported as the most frequently detected subtype although its proportion has steadily declined over the last years in favour of 3c [34]. No such drift is observed in Italy where 3f is reported as predominant [16]. In Bulgaria, HEV-3e is generally most frequently detected but locally restricted while 3f and to a lesser extent 3c, are scattered throughout the whole country [35]. Quite surprisingly, we did not detect rabbit HEV strains. Based on the range of detection frequencies (0.5–3.1%) reported from France, Spain and Switzerland [14,15,36,37], we would have expected to find at least some rabbit strains in our study sample. Although we cannot rule out the existence of rabbit HEV infections in Germany, we conclude from our results that the frequency is probably lower than reported for the countries mentioned above. Within Germany, specific subtypes did not cluster geographically, which is in line with results from England and Wales and compatible with a foodborne zoonosis with a centralized production, supra-regional distribution and retail of the main vehicle, i.e. pork products [38]. In this context, it is worth noting that a higher number of samples were referred to the national consultant laboratory during spring and summer – possibly due to seasonal eating habits, e.g. more frequent barbecuing in warmer periods. A similar observation and conclusion was drawn by Healy et al. who screened a large cohort of European blood donors and also observed higher HEV incidence rates in the months of May–July [39].

Long-term trends in the distribution of subtypes have been described in few countries. A shift from HEV-3f to 3c was observed in France, Belgium and the Netherlands [11,12,15]. In England and Wales, a general increase of group 2 strains was observed and attributed to an increased awareness and diagnosis of milder courses of hepatitis E [17]. As of 2001, HEV is a mandatorily notifiable pathogen in Germany and since then, the annual number of reported cases increased continuously. The increase of the proportion of HEV-3 group 2 infections is likely due to increased awareness and testing or changes in the spectrum of circulating HEV-3 subtypes in the animal reservoir and pork products. With regard to the proportion of HEV-3efg, such changes are likely relevant to human health and could influence disease burden.

A study from Italy found no correlation between HEV-3 subtype and clinical manifestations [18]. However, due to the very small overall sample size, only six HEV-3c cases were compared to 8 HEV-3e and 21 HEV-3f cases. These results must be interpreted with great caution because of the inherent lack of statistical power. Other studies with significantly larger sample sizes from Belgium and France showed that infections with subtypes HEV-3e and -3f were associated with a higher hospitalization rate and/or fever [19,20]. Our data are in line with these findings and further suggest that also jaundice and upper abdominal pain are more frequent in HEV-3 group 1 infections. Moreover, infection with group 1 (compared to group 2) was associated with fatality due to hepatitis E (odds ratio: 7.61). Group 1 strains were also overrepresented among strains infecting more than one individual, suggesting frequent common source exposures. It might also underline the increased virulence and point to a higher transmissibility. A previous study revealed increased in vitro replication for HEV-3e (but not 3f) compared to HEV-3c [40]. It remains to be shown if certain mutations in the viral genome confer these clinically relevant and subtype-specific characteristics.

There are some limitations to this study. First, our sample relied on voluntary referrals initiated by local public health departments. We have no information about when the samples were taken during the course of disease. While we do not expect a significant selection bias in terms of coverage of circulating strains, we cannot rule one out. Furthermore, in absence of a generally accepted cut-off for HEV sequence variation, we defined a comparably stringent criterion for related cases with presumably common source exposure. We cannot exclude that this specific approach might have prevented us from identifying all possibly related cases.

In Europe, HEV is an underdiagnosed, zoonotic pathogen and seroprevalence rates range up to 52.5% [3,8]. In Germany, approximately 400,000 infections are assumed per year based on an epidemiological model [9]. We could show that increasing numbers of acute HEV infections are mostly attributed to HEV-3 group 2 strains, while the amount of group 1 infections remains on a constant level. However, we show that infections with subtypes HEV-3e and 3f are associated with a more severe course of disease and higher mortality compared to other HEV-3 subtypes. We recommend implementing a comprehensive molecular surveillance of hepatitis E, sequencing all incident cases, monitoring HEV in the food chain and routinely comparing sequences in order to detect outbreaks distributed in space and time and enabling authorities to find specific sources and vehicles of infection. Moreover, the risk of zoonotic transmission still needs to be better communicated to vulnerable patients (i.e. immunocompromised individuals).

## Supplementary Material

Supplemental Material

## References

[1] Smith DB, Izopet J, Nicot F, et al. Update: proposed reference sequences for subtypes of hepatitis E virus (species Orthohepevirus A). J Gen Virol. 2020;101(7):692–698. 32469300 10.1099/jgv.0.001435 PMC7660235

[2] Pallerla SR, Harms D, Johne R, et al. Hepatitis E virus infection: circulation, molecular epidemiology, and impact on global health. Pathogens. 2020;9(10):856. 10.3390/pathogens9100856 PMC7589794 33092306

[3] Faber M, Askar M, Stark K. Case-control study on risk factors for acute hepatitis E in Germany, 2012 to 2014. Eurosurveillance. 2018;23(19):1732. 10.2807/1560-7917.ES.2018.23.19.17-00469 PMC5954605 29766841

[4] Lee G-H, Tan B-H, Teo EC-Y, et al. Chronic infection with Camelid hepatitis E virus in a liver transplant recipient who regularly consumes camel meat and milk. Gastroenterology. 2016;150(2):355–357.e3. 26551551 10.1053/j.gastro.2015.10.048

[5] Velavan TP, Pallerla SR, Johne R, et al. Hepatitis E: an update on one health and clinical medicine. Liver Int. 2021;41(7):1462–1473. 33960603 10.1111/liv.14912

[6] Lhomme S, Marion O, Abravanel F, et al. Clinical manifestations, pathogenesis and treatment of hepatitis E virus infections. JCM. 2020;9(2):331. 10.3390/jcm9020331 PMC7073673 31991629

[7] Adlhoch C, Manďáková Z, Ethelberg SJ, et al. Standardising surveillance of hepatitis E virus infection in the EU/EEA: a review of national practices and suggestions for the way forward. J Clin Virol. 2019;120:63–67. 31590112 10.1016/j.jcv.2019.09.005 PMC6899520

[8] Wilhelm B, Waddell L, Greig J, et al. Systematic review and meta-analysis of the seroprevalence of hepatitis E virus in the general population across non-endemic countries. PLoS ONE. 2019;14(6):e0216826. 31173594 10.1371/journal.pone.0216826 PMC6555507

[9] Faber M, Willrich N, Schemmerer M, et al. Hepatitis E virus seroprevalence, seroincidence and seroreversion in the German adult population. J Viral Hepat. 2018;25(6):752–758. 29377436 10.1111/jvh.12868

[10] Ijaz S, Said B, Boxall E, et al. Indigenous hepatitis E in England and Wales from 2003 to 2012: evidence of an emerging novel phylotype of viruses. J Infect Dis. 2014;209(8):1212–1218. 24273173 10.1093/infdis/jit652

[11] Suin V, Klamer SE, Hutse V M, et al. Epidemiology and genotype 3 subtype dynamics of hepatitis E virus in Belgium, 2010 to 2017. Eurosurveillance. 2019;24(10):1800141. 10.2807/1560-7917.ES.2019.24.10.1800141 PMC6415497 30862337

[12] Hogema BM, Hakze-van der Honing RW, et al. Comparison of hepatitis E virus sequences from humans and swine, the Netherlands, 1998-2015. Viruses. 2021;13(7):1265. 34209729 10.3390/v13071265 PMC8310231

[13] Lhomme S, Abravanel F, Dubois M, et al. Temporal evolution of the distribution of hepatitis E virus genotypes in Southwestern France. Infect Genet Evol. 2015;35:50–55. 26218544 10.1016/j.meegid.2015.07.028

[14] Abravanel F, Lhomme S, El Costa H, et al. Rabbit hepatitis E virus infections in humans, France. Emerg Infect Dis. 2017;23(7):1191–1193. 28628452 10.3201/eid2307.170318 PMC5512490

[15] Nicot F, Jeanne N, Roulet A, et al. Diversity of hepatitis E virus genotype 3. Rev Med Virol. 2018;28(5):e1987. 29939461 10.1002/rmv.1987

[16] Sabato L de, Di Bartolo I, Lapa D, et al. Molecular characterization of HEV Genotype 3 in Italy at human/animal interface. Front Microbiol. 2020;11:9. 32117156 10.3389/fmicb.2020.00137 PMC7014918

[17] Oeser C, Vaughan A, Said B, et al. Epidemiology of Hepatitis e in England and Wales: a 10-year retrospective surveillance study, 2008-2017. J Infect Dis. 2019;220(5):802–810. 31107958 10.1093/infdis/jiz207

[18] Minosse C, Biliotti E, Lapa D, et al. Clinical characteristics of acute hepatitis E and their correlation with HEV Genotype 3 subtypes in Italy. Pathogens. 2020;9(10):832. 10.3390/pathogens9100832 PMC7650787 33050666

[19] Subissi L, Peeters M, Lamoral S, et al. Subtype-specific differences in the risk of hospitalisation among patients infected with hepatitis E virus genotype 3 in Belgium, 2010-2018. Epidemiol Infect. 2019;147:e224. 31364564 10.1017/S0950268819001122 PMC6625206

[20] Abravanel F, Dimeglio C, Castanier M, et al. Does HEV-3 subtype play a role in the severity of acute hepatitis E? Liver Int. 2020;40(2):333–337. 31837187 10.1111/liv.14329

[21] Li S, He Q, Yan L, et al. Infectivity and pathogenicity of different hepatitis E virus genotypes/subtypes in rabbit model. Emerg Microbes Infect. 2020;9(1):2697–2705. 33251979 10.1080/22221751.2020.1858178 PMC7781933

[22] Hriskova K, Marosevic D, Belting A, et al. Epidemiology of Hepatitis e in 2017 in Bavaria, Germany. Food Environ Virol. 2021;13(3):337–346. 33900549 10.1007/s12560-021-09474-0 PMC8379136

[23] Wichmann O, Schimanski S, Koch J, et al. Phylogenetic and case-control study on hepatitis E virus infection in Germany. J Infect Dis. 2008;198(12):1732–1741. 18983248 10.1086/593211

[24] Wenzel JJ, Preiss J, Schemmerer M, et al. Detection of hepatitis E virus (HEV) from porcine livers in Southeastern Germany and high sequence homology to human HEV isolates. J Clin Virol. 2011;52(1):50–54. 21742549 10.1016/j.jcv.2011.06.006

[25] Jothikumar N, Cromeans TL, Robertson BH, et al. A broadly reactive one-step real-time RT-PCR assay for rapid and sensitive detection of hepatitis E virus. J Virol Methods. 2006;131(1):65–71. 16125257 10.1016/j.jviromet.2005.07.004

[26] Garson JA, Ferns RB, Grant PR, et al. Minor groove binder modification of widely used TaqMan probe for hepatitis E virus reduces risk of false negative real-time PCR results. J Virol Methods. 2012;186(1-2):157–160. 22871672 10.1016/j.jviromet.2012.07.027

[27] Johne R, Plenge-Bonig A, Hess M, et al. Detection of a novel hepatitis E-like virus in faeces of wild rats using a nested broad-spectrum RT-PCR. J Gen Virol. 2010;91(Pt 3):750–758. 19889929 10.1099/vir.0.016584-0

[28] Pearson WR. Finding protein and nucleotide similarities with FASTA. Curr Protoc Bioinformatics. 2016;53:3.9.1–3.9.25. 27010337 10.1002/0471250953.bi0309s53 PMC5072362

[29] Katoh K, Standley DM. MAFFT multiple sequence alignment software version 7: improvements in performance and usability. Mol Biol Evol. 2013;30(4):772–780. 23329690 10.1093/molbev/mst010 PMC3603318

[30] Stamatakis A. RAxML version 8: a tool for phylogenetic analysis and post-analysis of large phylogenies. Bioinformatics. 2014;30(9):1312–1313. 24451623 10.1093/bioinformatics/btu033 PMC3998144

[31] Ruscher C, Faber M, Werber D, et al. Resurgence of an international hepatitis A outbreak linked to imported frozen strawberries, Germany, 2018 to 2020. Eurosurveillance. 2020;25(37):1900670. 10.2807/1560-7917.ES.2020.25.37.1900670 PMC7502883 32945256

[32] Hogema BM, Molier M, Sjerps M, et al. Incidence and duration of hepatitis E virus infection in Dutch blood donors. Transfusion. 2016;56(3):722–728. 26559806 10.1111/trf.13402

[33] Harvala H, Hewitt PE, Reynolds C, et al. Hepatitis E virus in blood donors in England, 2016 to 2017: from selective to universal screening. Eurosurveillance. 2019;24(10):1800386. 10.2807/1560-7917.ES.2019.24.10.1800386 PMC6415500 30862338

[34] Izopet J, Tremeaux P, Marion O, et al. Hepatitis E virus infections in Europe. J Clin Virol. 2019;120:20–26. 31536936 10.1016/j.jcv.2019.09.004

[35] Bruni R, Villano U, Equestre M, et al. Hepatitis E virus genotypes and subgenotypes causing acute hepatitis, Bulgaria, 2013-2015. PLoS ONE. 2018;13(6):e0198045. 29879148 10.1371/journal.pone.0198045 PMC5991722

[36] Rivero-Juarez A, Frias M, Lopez-Lopez P, et al. Hepatitis E 3ra genotype infection in people living with HIV in Spain. Front Microbiol. 2020;11:564486. 33716992 10.3389/fmicb.2020.564486 PMC7945038

[37] Sahli R, Fraga M, Semela D, et al. Rabbit HEV in immunosuppressed patients with hepatitis E acquired in Switzerland. J Hepatol. 2019;70(5):1023–1025. 30803864 10.1016/j.jhep.2019.01.025

[38] Said B, Ijaz S, Chand MA, et al. Hepatitis E virus in England and Wales: indigenous infection is associated with the consumption of processed pork products. Epidemiol Infect. 2014;142(7):1467–1475. 24054519 10.1017/S0950268813002318 PMC9151183

[39] Healy K, Freij U, Ellerstad M, et al. Evaluating the prevalence of hepatitis E virus infection in a large cohort of European blood donors, 2015-2018. J Viral Hepat. 2022. 10.1111/jvh.13682 PMC9545359 35499211

[40] Schemmerer M, Johne R, Erl M, et al. Isolation of subtype 3c, 3e and 3f-like hepatitis E virus strains stably replicating to high viral loads in an optimized cell culture system. Viruses. 2019;11(6):483. 10.3390/v11060483 PMC6632007 31141895

