# Supplementary material for: Molecular epidemiology and genotype-specific disease severity of hepatitis E virus infections in Germany, 2010–2019
Source: Emerg Microbes Infect. 2022 Jul 17;11(1):1754–63. doi: 10.1080/22221751.2022.2091479 (PMC9295818; doi:10.1080/22221751.2022.2091479)
Supplement: Supplemental Material [file TEMI_A_2091479_SM9056.docx]

| **Year** | **HEV-3 subtype / monophyletic clade** | | | | | |  |
| --- | --- | --- | --- | --- | --- | --- | --- |
|  | **3c** | **3f** | **3e** | **3i(-like)** | **3m** | **3abjk** | **Total** |
| 2010 | 9 | 5 | 5 | 1 | 0 | 0 | n=20 |
| 2011 | 19 | 6 | 3 | 0 | 0 | 1 | n=29 |
| 2012 | 6 | 3 | 1 | 0 | 0 | 2 | n=12 |
| 2013 | 26 | 5 | 5 | 4 | 0 | 1 | n=41 |
| 2014 | 33 | 14 | 16 | 2 | 0 | 3 | n=68 |
| 2015 | 45 | 17 | 7 | 2 | 0 | 2 | n=73 |
| 2016 | 105 | 27 | 13 | 5 | 0 | 4 | n=154 |
| 2017 | 130 | 19 | 23 | 5 | 2 | 4 | n=183 |
| 2018 | 106 | 13 | 13 | 3 | 3 | 2 | n=140 |
| 2019 | 156 | 22 | 8 | 9 | 3 | 0 | n=198 |
| **Total** | n=635 | n=131 | n=94 | n=31 | n=8 | n=19 |  |

Supplementary Table 1. Temporal distribution of HEV-3 subtypes and monophyletic clade 3abjk.


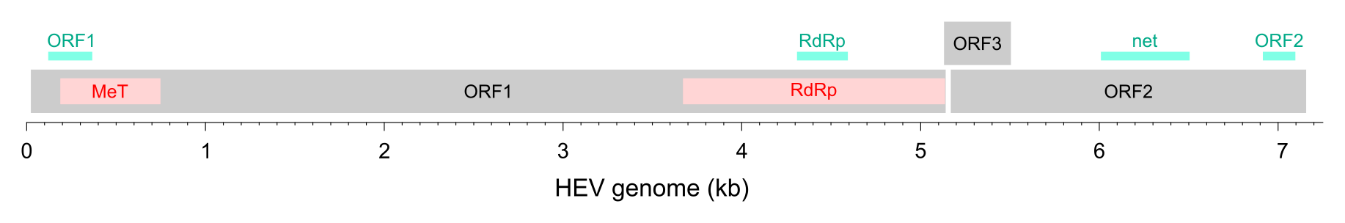


Supplementary Figure 1. Location of sequenced fragments (green) and overlapping functional domains (red) in relation to open reading frames (ORF). MeT, methyltransferase; net, HEVnet unified sequencing protocol fragment; RdRp, RNA-dependent RNA polymerase.


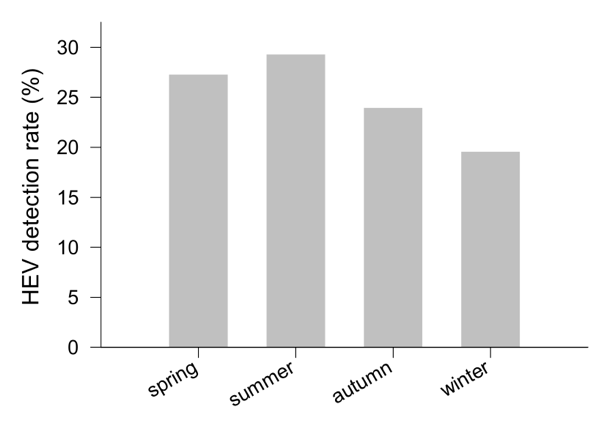


Supplementary Figure 2. Distribution of HEV PCR positive samples referred to the consultant laboratory by season, Germany, 2010–2019, n=936.


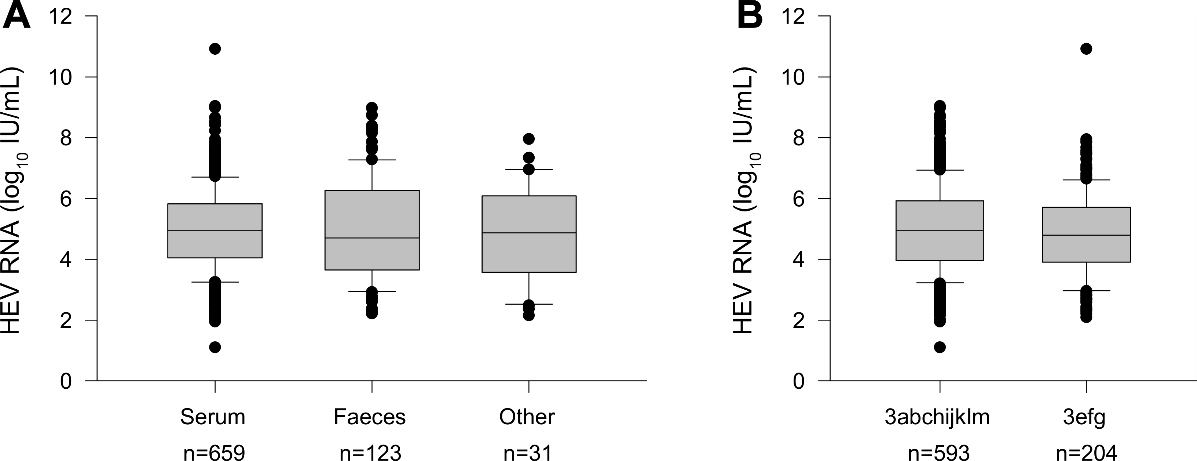


Supplementary Figure 3. Viral load (HEV RNA) in samples referred to the consultant laboratory by (A) sample material and (B) HEV-3 groups. Germany, 2010–2019; IU, international units.
